# What should patients do if they miss a dose? A systematic review of patient information leaflets and summaries of product characteristics

**DOI:** 10.1007/s00228-020-03003-x

**Published:** 2020-09-29

**Authors:** Abdullah Albassam, Dyfrig A. Hughes

**Affiliations:** 1 Department of Pharmacy Practice, Faculty of Pharmacy, Kuwait University, Kuwait City, Kuwait; 2 Centre for Health Economics and Medicines Evaluation, Bangor University, Bangor, LL57 2PZ UK

**Keywords:** Medication adherence, Labelling instructions, Patient information leaflets, Compliance

## Abstract

**Purpose:**

Medicines regulatory authorities advise that patient information leaflets (PILs) should provide specific advice on what actions to take if one or more doses are missed. We aimed to assess the content in this regard, of PILs and Summaries of Product Characteristics (SmPCs) of prescription only medicines (POMs) marketed in the UK.

**Methods:**

PILs and SmPCs were accessed via the electronic Medicines Compendium. The following terms were used in the advanced search facility: miss(ed), omit(ted), adhere(d), delay(ed), forgot, forget, lapse. Identified documents were screened for instructions on missed doses which were categorised according to level of specificity, and cross-referenced to the National Patient Safety Agency (NPSA) grading of risk of harm from omitted and delayed medicines. Any supporting clinical or pharmacological evidence was identified from SmPCs.

**Results:**

Two thousand two hundred eighty-four documents were identified from 7248 PILs and SmPCs relating to 1501 POMs. Seven hundred eighty-three (52%) POMs had SmPCs or PILs with no instructions on missed doses; 487 POMs (32%) included non-specific advice (e.g. “take as soon as possible”); 138 (9%) provided specific instructions; and 93 (6%) referred patients to seek medical advice. SmPCs for only 13/138 (9%) of those which included specific instructions provided any supporting clinical or pharmacological evidence. Instructions were absent for several medicines where the NPSA assessed that dose omissions may result in significant risk of harm.

**Conclusions:**

Advice on missed doses is generally inadequate. Pharmaceutical companies and regulatory authorities should produce clear and concise instructions on what patients should do if they miss doses, with supporting evidence where necessary.

## Introduction

Medication non-adherence is highly prevalent, impacts on patients’ quality of life and survival, and is costly to manage [1]. Adherence is a complex behaviour, which may be conceptualised in three parts: initiation (which occurs when the patient takes the first dose), implementation (the extent to which a patient’s actual dosing corresponds to the prescribed dosing regimen—from initiation until the last dose), and persistence (the time until the patient stops taking the prescribed medication) [2]. A common concern of patients who variably implement their prescribed medicine is what to do when a regular dose is occasionally delayed or missed [3].

Strict adherence to prescribed medications is important for achieving therapeutic outcomes, particularly for medicines which are less “forgiving”, that is, medicines that are associated with a sudden loss of therapeutic effect when doses are missed [4]. These drugs are typically associated with a short elimination half-life and/or have a rapid offset of action in relation to the dosing interval. Lapses in dosing therefore result in sub-therapeutic plasma concentrations and periods of insufficient pharmacologic activity. The often-held assumption that ≥ 80% doses taken represents adequate adherence is flawed on the basis that drug forgiveness is a function of a drug’s pharmacokinetic and pharmacodynamic properties and is therefore drug-specific [5]. Given that drug forgiveness is highly variable among different treatments, tailored instructions regarding the required action if a dose is delayed or omitted are therefore important [6, 7].

A review of drug labelling in the USA in 2000 found that 58 of the 76 package inserts assessed carried no information on what patients should do if doses are missed. Adequate information was provided in only 8 (11%) of cases [8]. The European Commission guideline on the preparation of Summaries of Product Characteristics (SmPCs) notes that where appropriate, advice should be provided on the “action to be taken if one or more dose(s) is (are) missed” [9]. The advice should be as specific as possible, taking into consideration the recommended frequency of dosing and relevant pharmacokinetic data. Patient information leaflets (PILs) have been a legal requirement as package inserts for all medicines in the UK since 1999. The Medicines and Healthcare products Regulatory Agency advises that information is provided on “what to do if a dose is missed” [10]. However, this is not mandatory and the extent to which PILs provide useful information for patients is unclear [11].

The aim of the present study was to systematically assess the PILs and SmPCs of prescription-only medicines with marketing authorisation in the UK to examine the extent to which they provide appropriate guidance on missed doses to patients and healthcare professionals.

## Methods

We systematically assessed the availability and detailed the content of instructions for missed doses in the PILs and SmPCs of all licensed prescription-only medicines catalogued in the UK electronic Medicines Compendium [12]. The advanced search feature was used to screen PILs and SmPCs for missing dose information by using the following keywords: miss(ed), omit(ted), adhere(d), delay(ed), forgot, forget, lapse.

All available PILs and SmPCs were included in the study and analysed further if any of the search terms pertained to instructions on what action should be taken if a dose is delayed or missed (herein denoted as “instructions for missed doses”). PILs and SmPCs were excluded if they were duplicates (e.g. multiple generic medicines), or related to different strengths of the same medicine.

Data on the following items were extracted from documents which were deemed relevant for inclusion: name of medicine (brand and generic); date of first marketing authorization; dosage form (oral, oral modified release, parenteral, topical, eye, ear, inhalation, “other”); dose frequency (less frequent than once daily, once daily, twice daily, three times daily, more than three times daily); source of information (PIL or SmPC); instructions for missed doses; and whether these instructions were supported by evidence.

All identified instructions for missed doses were categorised according to the level of detail of the instructions provided. We defined the following categories: no information, referral advice (e.g. “contact your doctor”), generic statement (e.g. “take it as soon as possible”), and specific instructions (e.g. as specified in an example provided by the European Medicines Agency [13], below):

“If the patient misses a dose of active substance X within 6 h of the time it is usually taken, the patient should be told to take it following a meal as soon as possible and then take the next dose at the regularly scheduled time. If a patient misses a dose by more than 6 h of the time it is usually taken, the patient should be told not to take the missed dose and simply resume the usual dosing schedule”

The association between the level of detail of the instructions for missed doses provided, and the decade of first marketing authorization, was tested using a chi-square test.

Instructions for missed doses in PILs and SmPCs were further assessed with reference to a tool produced by the UK National Patient Safety Agency (NPSA), which is aimed to reduce harm from omitted and delayed medicines in UK hospitals [14]. The tool does not list individual medicinal products, but rather, is structured according to British National Formulary (BNF) legacy chapters and sub-chapters, with each therapeutic class categorised using a traffic light system for the risk of harm from a delay or dose omission (Table 1). These risk categories are assigned to each of three scenarios: (i) dose not given at the time prescribed, (ii) dose not given within 2 h of time prescribed, and (iii) dose omitted (i.e. not administered by the time of next scheduled dose). For the present study, the highest risk category assigned by the NPSA was selected for each sub-chapter and was assumed to apply to the medicines contained therein.

**Table 1:** NPSA categorisation of risk of harm from omitting or delaying* a dose of medicine [14]

Risk category	Definition
Green: Nil or negligible patient impact with nil or minor intervention required; no increase in length of stay	• No or negligible risk of patient impact • No or minor intervention necessary • No possibility of an increase in the length of hospital stay
Amber: Significant short-term patient impact with moderate intervention required; increase in length of hospital stay possible	• Risk of significant short-term patient impact (i.e. significant loss of therapeutic effect, symptom control, or drug withdrawal effects) • Subsequent moderate intervention is required • Resultant long increase (1–15 days) in the length of hospital stay is possible
Red: Significant or catastrophic long-term patient impact with ongoing intervention required; long increase in length of stay possible	• Risk of significant long-term patient impact (i.e. the incident, and not the natural progression of illness or underlying condition, could permanently lessen bodily functions be they sensory, motor, physiological or intellectual) • Risk of catastrophic patient impact (i.e. death or severe irreversible health effects) • Subsequent ongoing professional intervention is required • Resultant very long (> 15 days) increase in the length of hospital stay is possible

*The omission or delay characteristics considered for each drug or drug class are as specified below:

a) Dose not given at the time prescribed

b) Dose not given within 2 h of time prescribed

c) Dose omitted (i.e. not administered by the time of the next scheduled dose)

Medicines were subsequently mapped onto NPSA risk categories to assess the relationship between labelling and the level of potential harm. To facilitate this mapping exercise, the Prescription Cost Analysis was used as a database of the belonging of each medicine to a BNF sub-chapter. Limitations to this approach were that the NPSA does not include all BNF sub-chapters; and neither does the Prescription Cost Analysis include all POMs.

The study was undertaken in April 2018 and is reported in accordance with the ESPACOMP Medication Adherence Reporting Guideline (EMERGE) [15].

## Results

**Fig. 1:**
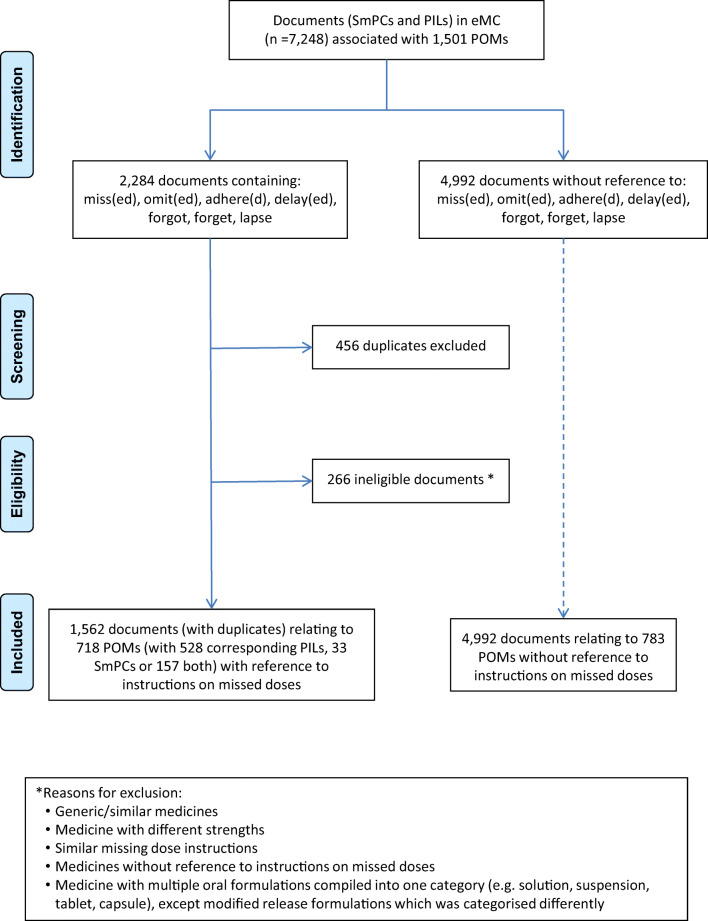
Flow diagram of search results

The search yielded 2284 documents from 7248 SmPCs and PILs relating to 1501 prescription only medicines listed in the electronic Medicines Compendium [12]. Following de-duplication, 783 (52%) POMs had SmPCs or PILs with no instructions on missed doses. A total of 685 PILs and 190 SmPCs relating to 718 unique POMs contained some form of instructions (Fig. 1). For 93 POMs (representing 6% of all POMs), these were referral advice; 487 (32% of POMs) had generic statements; and 138 (9% of POMs) had specific instructions (Table 2).

**Table 2:** Characteristics of the POMs and data sources analysed

Characteristics of results	Number (%)
Instructions for missed doses (out of all 1501 POMs)	
No instructions	783 (52)
Referral statements	93 (6)
Generic statements	487 (32)
Specific instructions	138 (9)
Source of information (out of 718)	
PIL	528 (73)
PIL and SmPC	157 (22)
SmPC	33 (5)
Dosage form (out of 718)	
Oral (immediate release)	450 (62)
Parenteral	144 (20)
Inhalation	30 (4)
Oral (modified release formulation)	29 (4)
Sensory (Eye and ear)	29 (4)
Topical	24 (3)
Other	12 (2)
Dosage frequency (out of 718)	
Less than once daily	86 (12)
Once daily	342 (48)
Twice daily	165 (23)
Three times daily	86 (12)
More than 3 times daily	39 (5)

Among medicinal products with instructions for missed doses, the majority (450/718; 62%) were oral formulations, followed by parenteral formulations (20%), and oral modified release formulations (4%) (Table 2). Treatments were mainly for once daily dosing (342/718; 48%). There was moderate evidence for a relationship between the decade of marketing authorization and the level of instruction on missed doses (Chi^2^ = 21.3; p = 0.046).

The NPSA tool includes 56 BNF legacy sub-chapters that covered 596 of the 718 POMs which included instructions on missed doses; and 134 of the 783 POMs that did not. Among the medicines identified as having instructions on missed doses, 235/718 (33%), 138/718 (19%), 223/718 (31%) were categorised as “red”, “amber”, and “green” according to the NPSA report, respectively.

There was no consistent inclusion of instructions for missed dose in PILs and SmPCs of medicines designated by the NPSA as having high or moderate risk of adverse outcomes when doses are delayed or omitted (Table 3). Among medicines within NPSA categories classed as “red”, 103/338 (25%) had no instructions for missed dose; 31/169 (20%) of medicines classed as “amber” had no instructions for missed doses, while 0/223 classed as “green” had no instructions for missed doses.

**Table 3:** Level of detail provided in instructions on missed doses for NPSA moderate/high risk category POM medicines. Data (number of medicines) are presented by BNF sub-section, with corresponding NPSA categorisation for the highest risk drug within each sub-section

BNF legacy chapter sub-section	No instruction	Referral statement	Generic statement	Specific instruction	NPSA risk category
Chapter 1: Gastro-intestinal system					
Antisecretory drugs and mucosal protectants	2	0	5	0	Amber
Chronic bowel disorders	1	0	10	0	Amber
Chapter 2: Cardiovascular system					
Positive inotropic drugs	1	0	2	0	Red
Diuretics	12	1	13	0	Red
Anti-arrhythmics	1	2	6	0	Red
Beta-adrenoceptor antagonists	5	0	13	0	Amber
Antihypertensives and heart failure	1	0	5	0	Red
Nitrates, calcium channel blockers, other antianginal drugs	1	0	5	1	Red
Sympathomimetics	3	0	0	0	Red
Anticoagulants and protamine	5	2	5	3	Red
Antiplatelet	3	0	4	1	Red
Stable angina, acute coronary syndrome, fibrinolysis	3	0	0	0	Red
Antifibrinolytics and hameostatics	0	0	1	0	Red
Chapter 3: Respiratory system					
Bronchodilators	0	0	22	0	Red
Corticosteroids	0	0	6	0	Red
Antihistamines, hyposensitisation and allergic emergencies	5	2	14	0	Red
Chapter 4: Central nervous system					
Anxiolytics	14	0	4	2	Red
Drugs used in psychoses	3	4	21	2	Amber
Analgesics	13	2	19	5	Red
Antiepileptic drugs	4	4	19	3	Red
Parkinsonism drugs	4	0	19	3	Red
Drugs used in substance dependence	0	2	8	1	Amber
Drugs for dementia	2	0	3	0	Amber
Chapter 6: Endocrine system					
Short acting insulins	0	0	4	0	Red
Intermediate- and long-acting insulins	2	0	7	0	Amber
Oral antidiabetic	1	0	28	6	Amber
Treatment of hypoglycaemia	0	0	1	0	Red
Replacement corticosteroids therapy	0	0	1	0	Red
Glucocorticoid therapy	2	2	6	0	Amber
Posterior pituitary hormones and antagonists	0	1	1	0	Red
Drugs affecting gonadotrophins	0	0	5	0	Red
Chapter 7: Obstetrics, gynaecology, and urinary-tract disorders					
Prostaglandins and oxytocics	2	1	0	0	Red
Ductus arteriousus	1	0	0	0	Red
Mifepristone	1	0	0	0	Red
Myometrial relaxants	2	0	0	0	Red
Emergency contraception	0	0	0	2	Red
Drugs used in urological pain	4	0	0	0	Amber
Bladder instillations and urological surgery	1	0	0	0	Amber
Chapter 8: Malignant disease and immunosuppression					
Alkylating agents, anthracyclines and other cytotoxic antibiotics, antimetabolites, other antineoplastic drugs	1	5	7	1	Red
Vinca alkaloids and etoposide	6	1	0	0	Red
Drugs affecting the immune response	0	0	7	0	Red
Sex hormones and hormone antagonists in malignant disease-depot preparations	1	4	12	2	Red
Chapter 9: Nutrition and blood					
Anaemias and some other blood disorders	0	4	2	0	Amber
Fluids and electrolytes	0	1	4	0	Red
Minerals	5	0	8	0	Red
Vitamin B group	2	0	0	0	Red
Vitamin K	2	0	1	1	Red
Chapter 10: Musculoskeletal and joint diseases					
Local corticosteroid injections	4	0	0	0	Amber
Drugs for rheumatic disease	0	1	12	2	Amber
Gout and cytotoxic-induced hyperuricaemia	3	0	3	0	Amber
Chapter 11: Eye					
Corticosteroids	5	0	16	0	Red
Mydriatics and cycloplegics	2	0	2	0	Red
Treatment of glaucoma	2	0	19	2	Red
Miscellaneous ophthalmic preparations	1	0	7	0	Red
Chapter 13: Skin					
Preparations for eczema and psoriasis	1	0	1	0	Amber
Sunscreens and camouflagers	0	0	2	0	Amber

Among licensed cardiovascular medicines, a substantial number of diuretics (12/25; 48%) and beta-adrenoceptor antagonists (5/18; 27%) had no instructions for missed doses. Oral antidiabetic medicines are categorised as amber risk, however, less than one fifth (6/35; 17%) had specific instructions for missed doses.

Among respiratory medicines, the PILs of all bronchodilators (22/22; 100%) and inhaled corticosteroids (6/6; 100%) had generic statements (e.g. administer as soon as possible). The PILs of 80% of medicines for rheumatic disease, which are amber NPSA risk category, also only included generic statements for missed doses. Neuropsychiatric medicines for psychoses, parkinsonism, and epilepsy, and which are also in the amber risk category, had generic statements for missed doses in 21/31 (67%), 19/30 (63%), and 19/26 (73%) of cases, respectively.

Among selected, specialist hospital medicines, all prostaglandins and oxytocics had either no or generic statements (i.e. contact your doctor); yet, the NPSA classed these drugs in the red risk category. Similarly, the majority of instructions for missed doses for cytotoxic agents are generic statements (13/14; 92%), even though the NPSA report classes anti-cancer medicines in the highest risk category.

We found evidence on pharmacokinetic and pharmacodynamic relationships with adherence in the SmPCs of 13/138 (9%) POMs that had specific instructions on missed doses (Table 4).

**Table 4:** Clinical and pharmacological information relating to duration of effect or to missed doses, as presented in the SmPCs

Drug name	Information on duration of effect or relating to missed doses
Acenocoumarol	The anticoagulant effect persists beyond 24 h
Amiodarone	It is strongly protein bound and has an average plasma half-life of 50 days (reported range 20–100 days). It follows that sufficient time must be allowed for a new distribution equilibrium to be achieved between adjustments of dosage. In patients with potentially lethal arrhythmias the long half-life is a valuable safeguard, as omission of occasional doses does not significantly influence the overall therapeutic effect. It is particularly important that the minimum effective dosage is used and the patient is monitored regularly to detect the clinical features of excess amiodarone dosage. Therapy may then be adjusted accordingly.
Atenolol	Effective for at least 24 h after a single oral dose. The drug facilitates compliance by its acceptability to patients and simplicity of dosing.
Atenolol, chlortalidone	It is effective for at least 24 h after a single oral daily dose. This simplicity of dosing facilitates compliance by its acceptability to patients.
Ethinylestradiol, norelgestromin	Results from an EVRA study of extended wear of single contraceptive transdermal patch for 7 days and 10 days indicated that target Css of norelgestromin and ethinyl estradiol were maintained during a 3-day period of extended wear of EVRA (10 days). These findings suggest that clinical efficacy would be maintained even if a scheduled change is missed for as long as 2 full days
Human normal immunoglobulin	Simulation of 2–3 missed daily doses resulted in a median serum IgG level decrease of ≤ 4% compared with consistent daily dosing. By replacing the missed doses when daily dosing was resumed, the median concentration profile recovered within 2 to 3 days. However, if missed doses were not replaced when dosing was resumed, it took up to 5–6 weeks for the IgG trough levels to return to steady-state
Insulin degludec	On occasions when administration at the same time of the day is not possible, Tresiba allows for flexibility in the timing of insulin administration. A minimum of 8 h between injections should always be ensured. There is no clinical experience with flexibility in dosing time of Tresiba in children and adolescents. Patients who forget a dose are advised to take it upon discovery and then resume their usual once-daily dosing schedule
Leuprorelin	The therapy is a long-term treatment, adjusted individually. PROSTAP 3 should be administered as precisely as possible in regular 3-monthly periods. An exceptional delay of the injection date for a few days (90 ± 2 days) does not influence the results of the therapy
Levonorgestrel, ethinylestradio	The efficacy of combined oral contraceptives may be reduced, in the event of missed tablets, vomiting or diarrhoea, or concomitant medication
Neostigmine	The usual duration of action of a dose is 2 to 4 h
Perampanel	Single missed dose: As perampanel has a long half-life, the patient should wait and take their next dose as scheduled. If more than 1 dose has been missed, for a continuous period of less than 5 half-lives (3 weeks for patients not taking perampanel metabolism-inducing anti-epileptic drugs (AED), 1 week for patients taking perampanel metabolism-inducing AEDs), consideration should be given to re-start treatment from the last dose level. If a patient has discontinued perampanel for a continuous period of more than 5 half-lives, it is recommended that initial dosing recommendations given above should be followed.
Terbutaline	The duration of action of a single dose is up to 6 h
Valsartan hydrochlorothiazide	The antihypertensive effect is substantially present within 2 weeks. In most patients, maximal effects are observed within 4 weeks. However, in some patients, 4–8 weeks treatment may be required. This should be taken into account during dose titration.

## Discussion

This is the first study to systematically assess the extent and nature of instructions for missed doses for all UK prescription only medicines. The study found that for the majority of medicines, there were no instructions for missed doses in either PILs or SmPCs. Moreover, where instructions were provided, most were generic, and probably inadequate to be informative to patients or healthcare professionals.

Given the high prevalence of variable implementation among ambulatory patients and of unintended delayed or omitted doses in hospitalised patients, the absence of instructions and widespread use of unspecific instructions provided to patients, prescribers and pharmacists in PILs and SmPCs is concerning. Patients recognising an error following self-administration of a medicine, and seeking information and reassurance may be complacent if no instructions are provided in the PIL, and this may lead to harm, especially if the medicine is unforgiving. Similarly, medication administration errors are a common occurrence on inpatient hospital wards [16, 17], and knowing what action to take is important for rectifying such errors. The observation that PILs and SmPCs for over a half of all POMs contained no instructions for missed doses is alarming, particularly as many of these are considered to be in high-risk categories by the NPSA.

Harm from delayed or omitted doses can result from reduced effectiveness or onset of adverse events, and is a function of a medicine’s forgiveness, efficacy and clinical indication. Manufacturers are not required to submit evidence on drug forgiveness to regulators, but while SmPCs provide essential data on the clinical pharmacology and adverse reactions of medicines, prescribers may not be sufficiently well informed to make specific recommendations to patients who enquire about missed doses. The extent to which medicines are forgiving to missed doses cannot be assumed from their pharmacokinetics, as forgiveness is also a function of drug pharmacodynamics [4, 5]. Aspirin, as one example, has a short elimination half-life, in the order of 2–3 h. However, the duration of antiplatelet effect does not correlate with the presence of salicylic acid in the circulation. Aspirin’s irreversible acetylation of platelet cyclo-oxygenase, coupled with the inability of platelets to synthesise new cyclo-oxygenase, means that the post-dose duration of action (forgiveness) is 7–10 days [18]. Missing occasional doses is largely inconsequential. Rivaroxaban and edoxaban have a longer plasma half-life (~ 12–14 h), but a rapid offset of effect (~ 1 day) owing to reversible inhibition of factor Xa which is closely correlated with their plasma concentrations. These are less forgiving to delayed and missed doses, and more likely to result in under anticoagulation and the associated risk of thrombosis in patients who poorly implement their dosing [7]. The SmPC for rivaroxaban [19]—but not edoxaban [20]—includes clear and specific instructions on missed doses. However, the details provided in the PILs for both drugs are very limited.

Among the SmPCs which provided explicit instructions for missed doses, some made reference to simulation, pharmacokinetic or clinical studies that provided supportive evidence. A range of methods are available to inform strategies on mitigating the effects of missed doses. Clinical trials designed to estimate the post-dose duration of action include substituting a single (or serial) dose(s) with placebo(s). In the case of oral contraceptives, this was done to define the interval between a last-taken pill before the ovulation-inducing surge of pituitary gonadotrophins appeared [21]. Similar studies have been performed with antihypertensive drugs [22–30] which indicate, for instance, amlodipine to be more forgiving than either enalapril or diltiazem, and to be more forgiving when combined with olmesartan than with perindopril [31]. For warfarin, pharmacokinetic-pharmacodynamic models have been proposed to inform labelling recommendations [32], and population pharmacokinetic models have been used to simulate missed doses of eslicarbazepine in patients with partial-onset seizures [33]. Pharmacometric analyses have also been used for a range of psychoactive treatments [34], and ought to be performed routinely to provide evidence for PIL and SmPC instructions on missed doses; especially for less forgiving treatments.

In our study, less than one tenth of UK POMs provided specific instruction on missed doses. These included medicines for neuropsychiatric disorders, type 2 diabetes as well as oral contraceptives and anticoagulants. A previous study in USA, reviewed the package inserts of 168 medicines found that only 8% had adequate information regarding medication adherence, while the majority (58%) had no information at all [8]. Our findings, that 52% of medicines have no information regarding missing dose instruction are consistent, but also reveal no change in practice over two decades. However, we recognise that SmPCs may not be updated in several respects, and our analysis suggests that medicines with more recent marketing authorisation provide better information.

Our study benefited from being systematic and including all prescription only medicines indexed in the electronic Medicines Compendium. However, there were some limitations. Specifically, the eMC is not complete—not all pharmaceutical companies subscribe to Datapharm to publish their information on the eMC website, and so a small proportion of medicines licensed in the UK will not have been included. We were also constrained by the advanced search facility of the eMC, which provided limited flexibility for searching, especially within portable document format (pdf) files, which represented many PILs. Our search may also have lacked sensitivity through the omission of potentially relevant search terms. Nevertheless, we considered that the study was conducted robustly with sampling that was sufficiently reflective of UK licensed POMs without any systematic bias. Finally, the classification of medicines in the NPSA tool is based on consensus of the opinions of pharmacists and patient safety specialists, and validated by an expert panel. It focuses primarily on the use of medicines in hospitals with indications deemed of particular relevance for primary care not having been considered. It also does not have full coverage of BNF sub-chapters, and therefore excluded 771/1501 (51%) of eMC listed medicines. Most of these (649/771; 84%) had no instructions on missed doses, suggesting the NPSA tool may select for high risk medicines, as might be expected.

In conclusion, the high prevalence of non-adherence to medications, particularly in the form of variable implementation and its associated hazards, requires that patients and healthcare professionals need to be suitably informed on what actions to take when doses are delayed or missed. Patients often face a dilemma on what to do when they miss a dose, and while patient information leaflets and summaries of products characteristics are considered key references, the advice currently offered in these resources is largely inadequate. We recommend that further regulatory oversight is warranted, to ensure the safeguard of patients through the provision of appropriate information. This is especially important for less forgiving medicines, where specific trials or simulations may be necessary to provide the relevant evidence.

## Data Availability

Data can be supplied upon request.
